# Dedifferentiation of patient-derived glioblastoma multiforme cell lines results in a cancer stem cell-like state with mitogen-independent growth

**DOI:** 10.1111/jcmm.12479

**Published:** 2015-03-19

**Authors:** Inan Olmez, Wangzhen Shen, Hayes McDonald, Bulent Ozpolat

**Affiliations:** a Department of Neurology, Vanderbilt UniversityNashville, TN, USA; b Department of Biochemistry, Vanderbilt UniversityNashville, TN, USA; c Department of Experimental Therapeutics, The University of Texas MD Anderson Cancer CenterHouston, TX, USA; d Non-Coding RNA Center, The University of Texas MD Anderson Cancer CenterHouston, TX, USA

**Keywords:** glioblastoma multiforme, cancer stem cells, salinomycin, epidermal growth factor receptor, Oct4, Nanog, Sox2, EphB4

## Abstract

Emerging evidence shows that glioblastoma multiforme (GBM) originates from cancer stem cells (CSCs). Characterization of CSC-specific signalling pathways would help identify new therapeutic targets and perhaps lead to the development of more efficient therapies selectively targeting CSCs. Here; we successfully dedifferentiated two patient-derived GBM cell lines into CSC-like cells (induced glioma stem cells, iGSCs) through expression of Oct4, Sox2 and Nanog transcription factors. Transformed cells exhibited significant suppression of epidermal growth factor receptor and its downstream pathways. Compared with parental GBM cells, iGSCs formed large neurospheres even in the absence of exogenous mitogens; they exhibited significant sensitivity to salinomycin and chemoresistance to temozolomide. Further characterization of iGSCs revealed induction of NOTCH1 and Wnt/β-catenin signalling and expression of CD133, CD44 and ALDH1A1. Our results indicate that iGSCs may help us understand CSC physiology and lead to development of potential therapeutic interventions aimed at differentiating tumour cells to render them more sensitive to chemotherapy or other standard agents.

## Introduction

Glioblastoma multiforme (GBM) is the most common primary brain tumour and contains a heterogeneous population of cells, including cancer stem cells (CSCs) 1. There is growing evidence that anaplastic gliomas and GBMs originate from CSCs that eventually differentiate into a phenotypically diverse cell population 1–3. Similar to normal stem cells, CSCs exhibit asymmetric cell division to give rise to a daughter stem cell for unlimited self-renewal and a daughter progenitor cell that undergoes further differentiation that comprises the tumour mass 4. While the exact source and the mechanism of transformation remain to be defined, CSCs are thought to originate from either malignant transformation of tissue-specific stem/progenitor cells through genetic and epigenetic alterations or dedifferentiation of differentiated cells.

Cancer stem cells are highly resistant to conventional cancer therapy, leading to failure of therapeutic options, including chemotherapy and radiotherapy, and to tumour recurrence. Thus characterization of CSC-specific signalling pathways and identification of targetable markers constitute major steps forward in understanding CSC biology, isolating tumour-specific CSCs and developing more efficient targeted therapy. For glioma stem cells (GSCs), several markers have been proposed including CD133, CD44, L1CAM and SSEA-1 5–7. However, the functional significance and validity of these markers remain unclear. Emerging data indicate that developmentally preserved signalling pathways such as NOTCH and Wnt/β-catenin are aberrantly activated in GSCs and promote their survival 8,9. As in neural stem cells, these pathways are believed to be responsible for regulation of maintenance and differentiation of GSCs and to promote tumour growth 10,11 and confer resistance to cancer therapy 12,13.

Recently, reprogramming of normal somatic cells (adult human fibroblasts) into pluripotent stem cells through forced expression of Oct4, Nanog, Sox2, Lin28 and Klf4 was demonstrated 14. In our study, we utilized Oct4, Nanog and Sox2 transcription factors, which are linked to oncogenic transformation 15,16, and successfully reprogrammed two patient-derived GBM cell lines into CSC-like cells, which we term induced glioma stem cells (iGSCs). We subsequently compared these transformed cells to parental cells. iGSCs were pluripotent in nature, formed much larger neurospheres in a short period of time independently of exogenous growth factors and exhibited more resistance to temozolomide. We also identified significant molecular changes including suppression of epidermal growth factor receptor (EGFR), ERK, PI3K/AKT and its downstream signalling, and activation of NOTCH1 and β-catenin pathways, suggesting that iGSC may help us further understand the biology and regulation of CSC-like cells.

## Materials and methods

### Cell cultures of GBM cells and iGSCs

Two patient-derived GBM lines were obtained according to Vanderbilt University IRB protocols (VU numbers; GBM1: 18594 and GBM2: 18577). GBM cells and iGSCs were cultured on Matrigel-coated (BD Biosciences San Jose, CA, USA) six-well plates in attached form and fed daily with NeuroCult medium (Stem Cell Technologies Vancouver, BC, Canada) throughout the study. During early passage of GBM cells, a subgroup of cells were separated and cultured in medium containing 10% serum for further differentiation of GBM tumour–initiating cells. Two weeks later, these cells were transfected with plasmids. Dispase was used for passage of cells. The comparison of signalling pathway activation was performed between iGSCs and GBM cells cultured in stem cell medium.

### Reprogramming of GBM cells into iGSCs

Glioblastoma multiforme cells (500,000) from each line were collected and mixed with 1 μg of DNA from each plasmid carrying Oct4, Nanog and Sox2 transcription factors with the addition of shP53 and L-Myc (pCXLE-hSK, pCXLE-hUL and pCXLE-hOCT3/4-shp53-F; Addgene). This mixture was loaded into a special pipette that was then placed into a cuvette filled with electrical buffer. Transfection was completed with the Neon electroporation system (Invitrogen Grand Island, NY, USA) according to the manufacturer's protocol. Settings for electroporation were 1400 V, double pulse and 20 msec. width. Transfected cells were then seeded onto gelatin-coated six-well plates at 5000 cells per well. Cells were fed with DMEM/F12 medium every other day during the first week. Starting with the second week, medium was changed to hES medium [DMEM/F12, 20% Knockout serum replacement (Invitrogen), 2 mM Glutamax (Invitrogen), non-essential amino acids (Sigma-Aldrich St. Louis, MO, USA), penicillin/streptomycin (Mediatech Manassas, VA, USA), 55 μM β-mercaptoethanol (Sigma-Aldrich) and recombinant human FGF-2 (10 ng/ml, Promega Madison, WI, USA)] supplemented with leukaemia inhibitory factor (LIF; 10 ng/ml, Millipore Billerica, MA, USA). Approximately 3 weeks after transfection, individual colonies composed of smaller and round cells were identified among elongated and larger parental GBM cells. These colonies were separated from parental cells using a pipette and transferred to Matrigel-coated plates. Meanwhile, hES medium plus LIF was changed to mTeSR1 or NeuroCult medium within 2 days for further propagation of cells.

### Neurosphere formation

Both parental GBM cells and iGSCs were seeded at 50 cells per well in parallel onto six-well plates with an ultra-low attachment surface (Corning Tewksbury, MA, USA). Cells were cultured with hES medium with and without EGF (20 ng/ml). Half of the medium was replaced carefully with fresh medium every 2 days. After a week of culturing, large neurospheres were observed and live images were taken for comparison.

### Western blot, ELISA-based pathway analysis and immunocytochemistry

Cells were lysed in RIPA buffer containing 1% protease and phosphatase inhibitors and sonicated. This was followed by gel electrophoresis using the NuPAGE SDS-PAGE gel system (Invitrogen) and transfer to PVDF-FL membranes (Millipore). Primary antibodies were Oct-4, SOX2, EGFR, p-Akt, Akt, pS6 (Ser235/236), S6, p-Erk1/2, Erk1/2 (1:1000, Cell Signaling Boston, MA, USA), Nanog (1:500), Axin2 (1:1000; Abcam Cambridge, MA, USA), NOTCH1 (1:200), CD133 (1:500; Santa Cruz Biotechnology Dallas, TX, USA) and β-catenin (1:1000, Vanderbilt University antibody and protein resource). β-Actin (1:1000, Sigma-Aldrich) was used as control antibody. IRDye conjugated secondary antibodies (1:10,000, LI-COR) were used and the signal was visualized using Odyssey Imaging (LI-COR Lincoln, NE, USA).

Phosphorylation of various receptor tyrosine kinases (RTK) and their downstream signal transducers was detected with a Pathscan sandwich ELISA kit (Cell Signaling) according to the kit manual, with each analysis performed in quadruplicate. Fluorescent intensity was detected using Odyssey Imaging. After background subtraction, each value was normalized to positive controls. Further details are given in the study by Slanina *et al*. 17.

Immunocytochemistry was performed as described previously 18. The following primary antibodies were used: Oct-4, GFAP (1:200, Cell Signaling), ALDH1A1 (1:200), smooth muscle actin (1:50), GATA4 (1:200; Sigma-Aldrich), CD44 (1:400, Abcam) and β-III tubulin (1:200, Millipore).

### RNA extraction, cDNA synthesis and qRT-PCR

Total RNA was extracted from cells by using both Trizol (Invitrogen) and the RNeasy kit (Qiagen Valencia, CA, USA). After quantification using a NanoDrop (Thermo Scientific Waltham, MA, USA), cDNA was synthesized with the SuperScript VILO cDNA synthesis kit (Life Technologies Grand Island, NY, USA) according to the manufacturer's guidelines. Negative control reactions were performed without reverse transcriptase. qRT-PCR was performed with SYBR Green Supermix (Bio-Rad Irvine, CA, USA). Reactions for each sample were performed in triplicate with negative controls. Primer sequences were as follows: EGFR forward, 5′-TCCTCTGGAGGCTGAGAAAA-3′, EGFR reverse, 5′-GGGCTCTGGAGGAAAAGAAA-3′ 19, Oct4 forward, 5′-AAAGCGAACCAGTATCGAGAAC-3′, Oct4 reverse, 5′-GCCGGTTACAGAACCACACT-3′ and GAPDH forward, 5′-GCTCTCTGCTCCTCCTGTTC-3′, GAPDH reverse, 5′-CGTTGACTCCGACCTTCAC-3′ 18.

### Flow cytometry

For comparison of CD133 expression, flow cytometry was performed with phycoerythrin (PE)-conjugated CD133/1 clone AC133 antibody (1:50; Miltenyi Biotec San Diego, CA, USA). Cells were initially resuspended and washed twice with FACS buffer (1× PBS, 2% FBS, 0.05% sodium azide, 1 mM EDTA) followed by incubation with antibody for 1 hr on ice. After three washes, cells were resuspended in FACS buffer and analysed by flow cytometry. 7-Amino actinomycin D (7AAD) was used for exclusion of dead cells. For cell cycle analysis, cells were fixed in 70% ethanol and stained with propidium iodide according to kit instructions (Abcam). Signal was detected using FACSDiva software (BD Biosciences).

### Neural lineage formation and multilineage differentiation

Following treatment with Accutase cell detachment solution, 10–20 cells per microlitre were seeded onto six-well plates with an ultra-low attachment surface (Corning) and fed daily with hES medium for 4–6 days until embryoid body (EB)-like spheres were seen. For neuronal differentiation, EBs were collected and plated on gelatin-coated six-well plates containing hES medium. The next day, medium was changed to ITSF medium [DMEM/F12 supplemented with 5 μg/ml fibronectin (Sigma-Aldrich), insulin, transferrin and selenium solution (Life Technologies)] and attached colonies were fed daily for 5 days. ITSF medium was then changed to N2 medium for further propagation and maintenance of colonies.

For multilineage differentiation, EBs were placed on 24-well plates coated with gelatin and fed with hES medium without FGF-2 every other day for 4–6 days.

### Drug sensitivity assays

Parental GBM cells and iGSCs were seeded at 5000 cells per well onto 96-well plates and cultured in medium containing increasing concentrations of salinomycin (Sigma-Aldrich) for 2 days or temozolomide (Sigma-Aldrich) for 4 days. Untreated cells were used as controls. Four wells were used for each concentration group. After treatment, viability of cells was measured with the MTT assay 20.

### Statistical analysis

We used GraphPad Prism 5 software for statistical analysis and graphics formation. Each experiment was performed at least three times. Student's two-tailed *t*-test was used to compare GBM *versus* iGSC for statistical significance. *P* ≤ 0.05 were considered significant.

## Results

### Dedifferentiation of GBM lines into iGSCs

**Figure 1:**
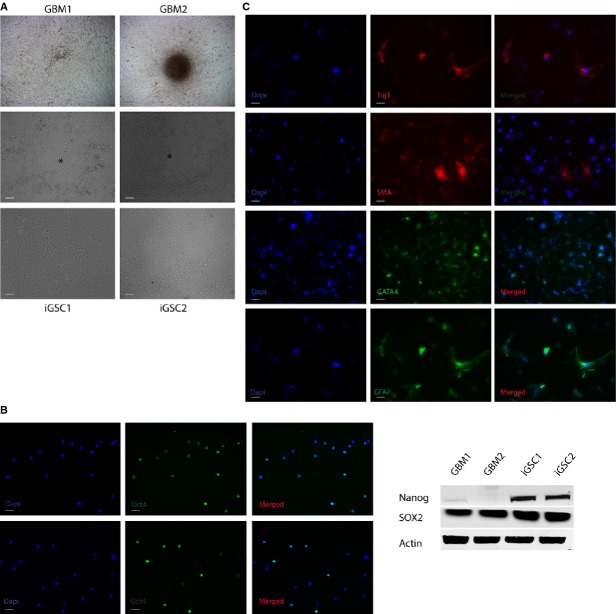
Dedifferentiation of glioblastoma multiforme (GBM) cell lines into induced glioma stem cells (iGSCs). (A) Shown are microscopic images of GBM cells (top), emerging colonies marked with asterisk (middle) and iGSCs (bottom). Scale bar: 100 μm. (B) Analysis of iGSC2 for pluripotency markers such as Oct4, Nanog and Sox2. Oct4 was detected with immunocytochemistry. DAPI was used for nuclear staining. Nanog and Sox2 expressions were compared using Western blot. Sox2 expression is 2.5-fold higher in iGSCs. Actin was used as control for Western blot. Scale bar: 100 μm. (C) Multilineage differentiation of iGSC2 detected with immunocytochemistry: Tuj-1 for ectoderm, GFAP for neuronal, GATA4 for endoderm and SMA for mesoderm. DAPI was used for nuclear staining. Scale bar: 100 μm.

To reprogram two patient-derived GBM cell lines into CSC-like cells, GBM1 and GBM2 were cultured and stably transfected with expression plasmids containing *Oct4, Nanog* and *Sox2* transcription factors (Fig.1A, top). The passage number for each line ranged from three to 15 throughout the study. Colonies emerged 2–3 weeks after transfection (Fig.1A, middle). Compared with parental cells, these transformed cells were round and smaller and exhibited a morphological appearance similar to that of stem cells. The colonies were manually selected based on morphology and named iGSC1 and iGSC2 (derived from GMB1 and GBM2, respectively; Fig.1A, bottom). As a confirmation of successful transfection, iGSCs were tested for transcription factors such as Oct4, Nanog and Sox2 (Fig.1B). We next evaluated the pluripotency potential of iGSCs. Interestingly, transformed cells could differentiate into different lineages (endoderm, ectoderm and mesoderm) through EB formation as indicated by expression of Tuj1, GFAP, SMA and GATA4 (Fig.1C, Fig. S1).

### Neural lineage formation and comparison of cell cycles

**Figure 2:**
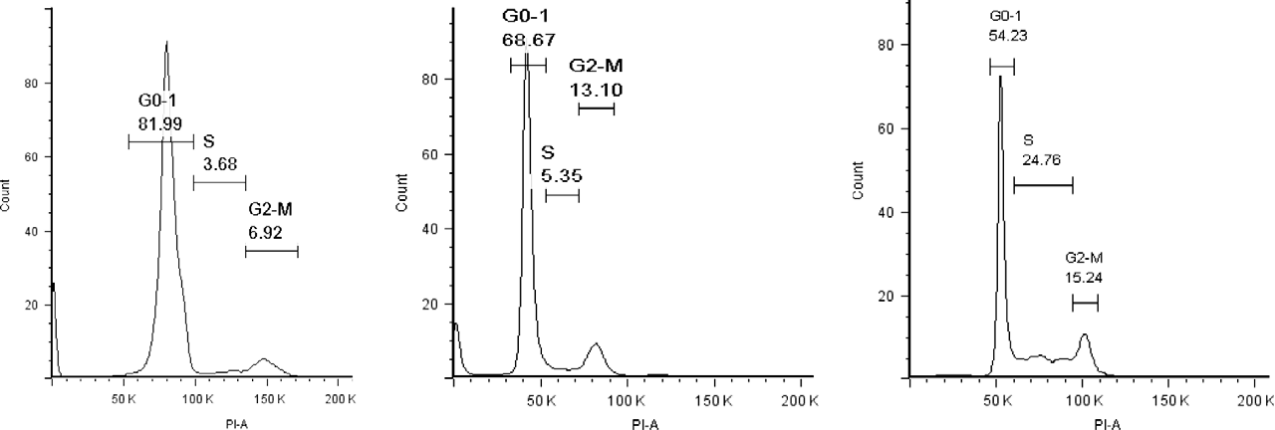
Cell cycle analysis, showing induced glioma stem cells (iGSCs) on the left, glioblastoma multiforme (GBM) in the middle and neuronally differentiated cells on the right. Upon differentiation, iGSC cells entered proliferative phase as indicated by a significant shift towards S/G2/M phase (*P* < 0.05). Compared with GBM cells, iGSCs were mainly in a dormant state.

Embryonic stem cells (ESC) ESCs mainly stay in a dormant state and enter proliferative phase based on various stimulations. Similarly, CSCs are thought to be in quiescent phase and enter proliferative phase upon differentiation. Because the ability to form tumour mass depends largely on CSC differentiation, we induced iGSCs to differentiate into neural lineage (Fig. S2) and compared their cell cycle profiles as an indirect measure of tumour formation potential. While iGSCs were mainly in the dormant state in comparison to GBM, we observed significant shift towards S/G2/M phase (81.99, 3.68 and 6.92 *versus* 54.23, 24.76 and 15.24) upon neuronal differentiation (Fig.2). This result may indicate that iGSCs have potential of entering proliferative phase and tumour formation upon differentiation.

### iGSCs form much larger neurospheres independently of exogenous mitogens

**Figure 3:**
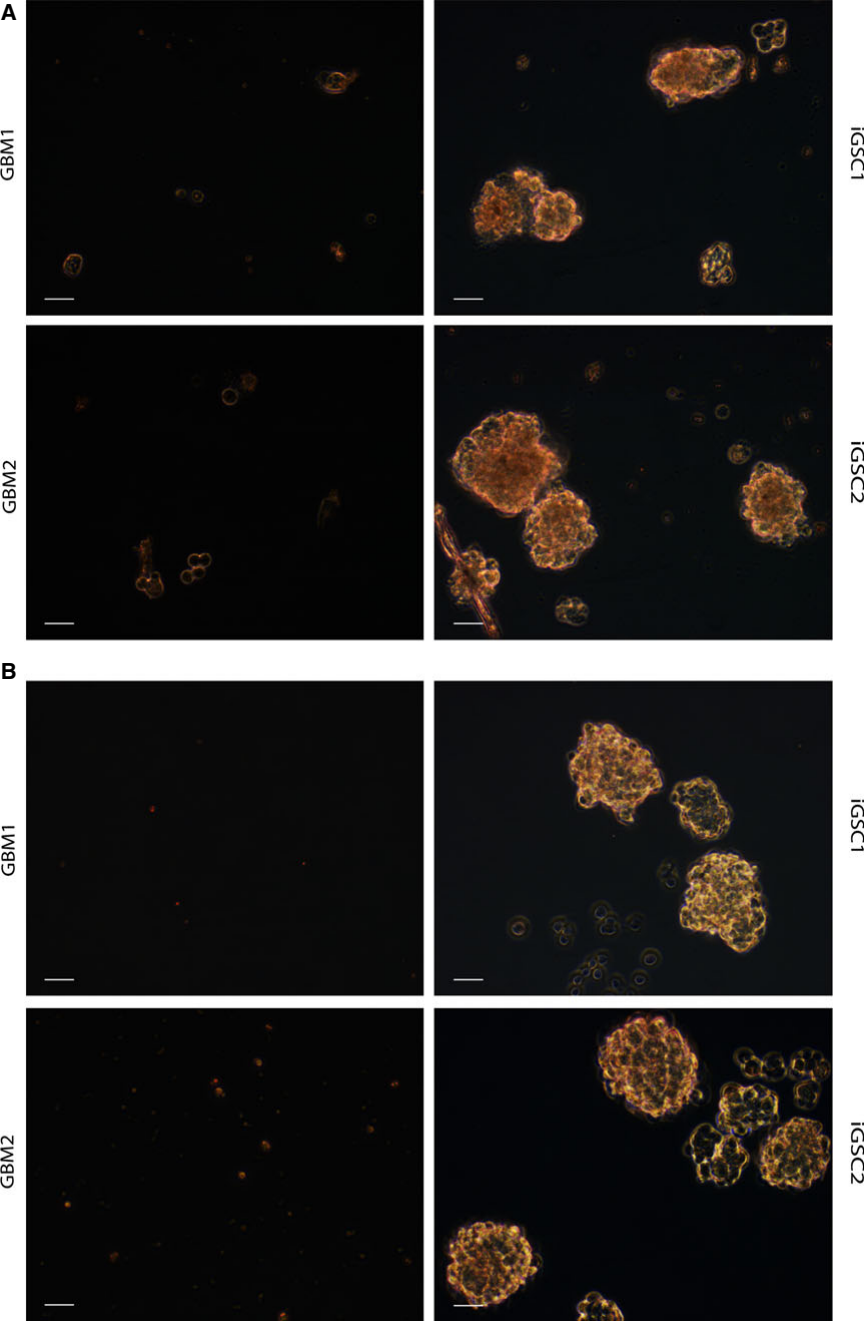
Neurosphere formation. (A) Induced glioma stem cells (iGSCs) formed larger neurospheres compared with glioblastoma multiforme (GBM) lines within a week with culture medium containing both EGF and FGF-2. Scale bar: 50 μm. (B) Although GBM lines could not survive in the absence of growth factors, iGSCs could still form large neurospheres. Scale bar: 50 μm.

Neurosphere formation assay has been utilized to evaluate the self-renewal and differentiation potential of brain tumour stem cells and shown to be an independent predictor of clinical outcome in malignant gliomas 21. Upon our initial experiment with medium containing EGF, iGSCs formed much larger neurospheres in a week compared with parental GBM cells (Fig.3A). Because stem cells could proliferate independently of exogenous mitogens 2, we repeated the assay in the absence of both EGF and FGF-2. While there was no change in the growth rate and sphere formation of iGSCs, parental GBM cells simply could not survive and form spheres (Fig.3B). These results indicate that iGSCs could turn on CSC-specific pathways that confer higher growth potential independently of exogenous mitogens.

### Epidermal growth factor receptor is significantly down-regulated in iGSCs

**Figure 4:**
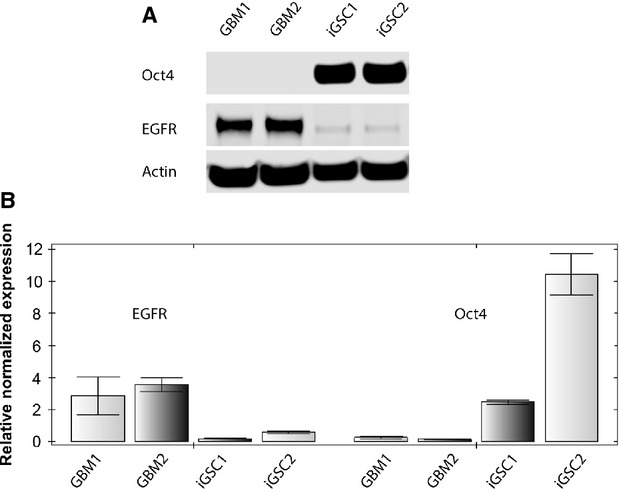
Suppression of epidermal growth factor receptor (EGFR) expression in induced glioma stem cells (iGSCs). EGFR expression was suppressed in iGSCs with acquisition of stem cell-like features, as detected by Western blot (A) and qRT-PCR (B). Unlike iGSCs, glioblastoma multiforme (GBM) cells were negative for Oct4 and had increased EGFR expression (*P* < 0.01). Actin and GAPDH were used as controls for Western blot and qRT-PCR, respectively.

To examine alterations in the signalling, we analysed several important pathways in iGSCs. EGFR amplification has been observed in about 50% of GBMs 22. Aberrant activity of this receptor promotes cell survival, invasiveness and resistance to therapy 23,24. As also seen with the neurosphere formation assay, GBM lines require external EGF supplementation for growth. On the basis of the above findings, we next compared EGFR expression levels of both parental cells and iGSCs and found a significant down-regulation of EGFR expression in iGSCs compared with parental GBM cells, as detected by Western blot analysis (Fig.4A). We further confirmed this difference with qRT-PCR (Fig.4B).

**Figure 5:**
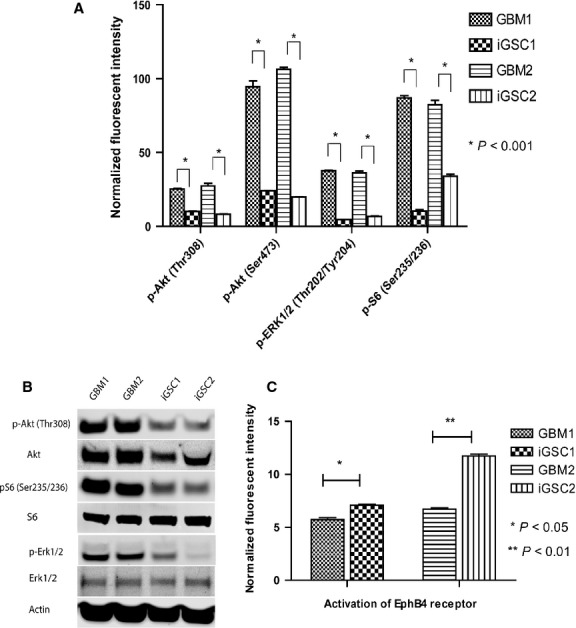
Receptor tyrosine kinases (RTK) and downstream pathways. (A and B) There was significantly decreased activity of p-Akt (Thr308), p-Akt (Ser473), p-S6 (Ser235/236) and p-Erk1/2 (Thr202/Tyr204) in induced glioma stem cells (iGSCs) compared with glioblastoma multiforme (GBM) cells, as detected by Western blot and ELISA (*P* < 0.001). (C) EphB4 receptors were significantly activated in iGSCs compared with GBM lines.

Epidermal growth factor receptor is a member of the Erb family RTK. RTKs are composed of various receptor families and are implicated in tumour growth. Upon phosphorylation, signal is transmitted to downstream signalling pathways such as MAPK and Akt, which subsequently stimulate tumour growth and invasion. Prominent differences were seen in downstream pathways involving Erk1/2 and Akt (Fig.5A and B). Upon screening of major RTKs, ephrin type-B receptor 4 (EphB4) RTK, which has a regulatory role in neural and vascular development, was found to be significantly activated in iGSCs (Fig.5C). The remaining major RTKs and downstream pathways were significantly inactive in iGSCs compared with parental cells (Fig. S3). Activities of PDGFR, STAT1 and STAT3 were highly down-regulated in iGSCs (Fig. S3).

### NOTCH1 and Wnt/β-catenin pathways are activated in iGSCs

**Figure 6:**
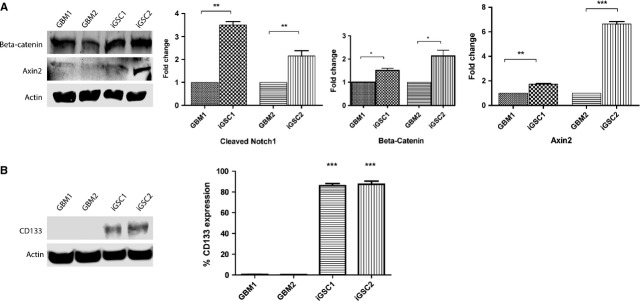
Activation of stem cell-specific pathways. (A) Western blot revealed activation of NOTCH1, β-catenin and Axin2. Bar graphics show fold changes in fluorescent intensities normalized to control (actin; *P < 0.05, **P < 0.01, ***P < 0.001). (B) Unlike glioblastoma multiforme (GBM) cells, induced glioma stem cells (iGSCs) were positive for CD133, as detected by Western blot (left). On the right, flow cytometry shows the per cent population of CD133^+^ cells. Although GBM1 and GBM2 cells were negative, 86% of iGSC1 and 87% of iGSC2 cells were positive for CD133 (***P < 0.001).

Because dedifferentiation of GBM cells resulted in acquisition of stem cell-like features, we studied the state of key pathways involved in CSC survival and maintenance such as the NOTCH and Wnt/β-catenin pathways, which also mediate neural developmental processes. We found that NOTCH1 and β-catenin were activated in iGSCs compared with GBM cells (Fig.6A). Because of the association of CD133 with NOTCH1 activity, we also checked CD133 status. While parental GBM cells were negative for CD133, with dedifferentiation of GBM cells we observed conversion into the CD133^+^ state (Fig.6B). In addition, similar to previous reports, our iGSCs were positive for CD44 and ALDH1A1 (Fig. S4).

### iGSCs are more resistant to conventional cancer drugs and more sensitive to salinomycin

**Figure 7:**
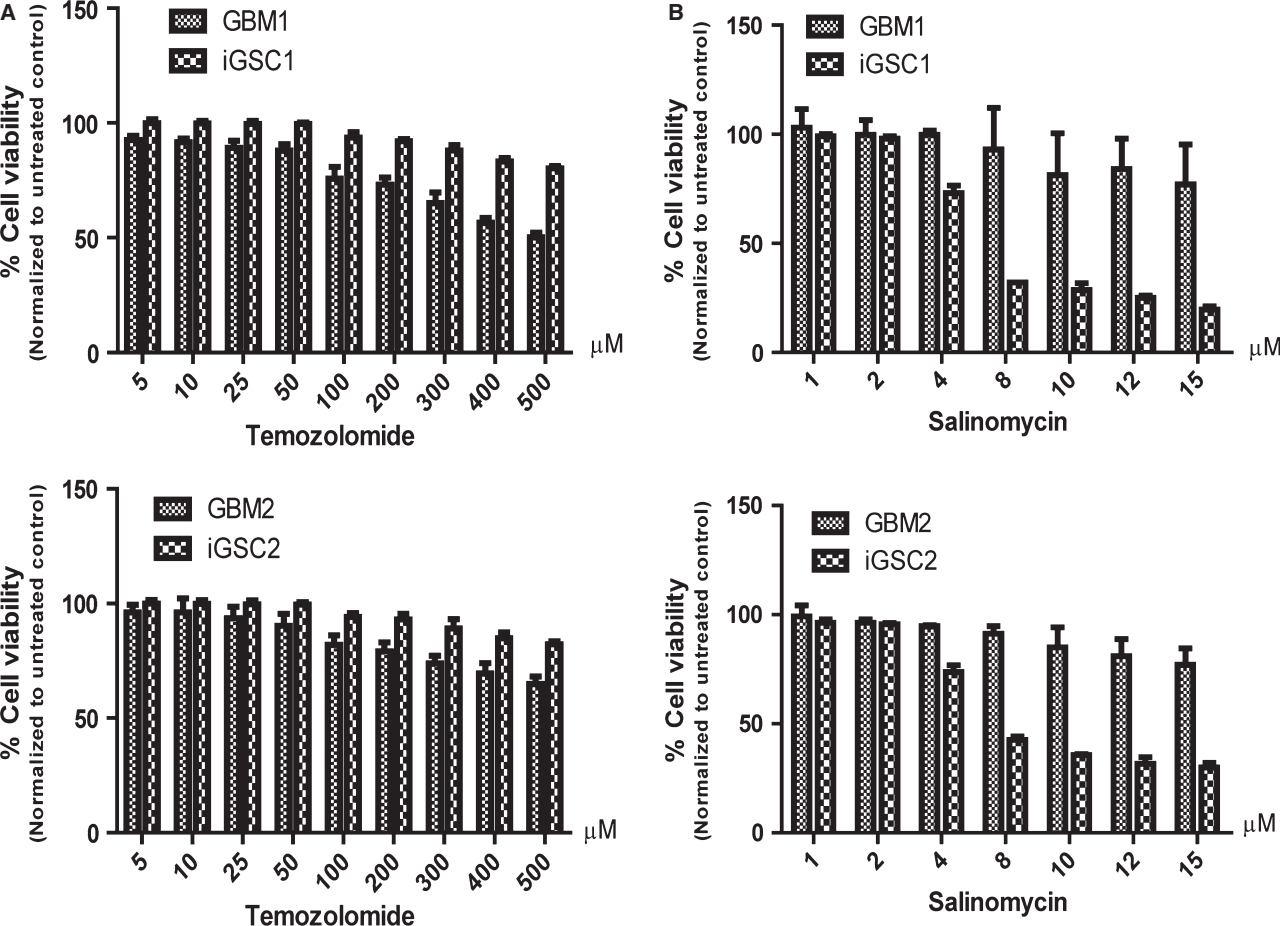
Drug sensitivity analysis. (A) Induced glioma stem cells (iGSCs) were more resistant to 4-day temozolomide treatment than glioblastoma multiforme (GBM) lines (*P* < 0.05). (B) Compared with GBM lines, iGSCs were highly sensitive to 2-day salinomycin treatment (*P* < 0.01). Cell viability was detected by using the MTT assay.

To further characterize iGSCs, we compared their response to different cancer drugs. Temozolomide is the most important agent used for treatment of GBM. Upon 4-day treatment with increasing doses of temozolomide, survival rates were significantly higher for iGSCs than for GBM cells (Fig.7A). Actinomycin D is commonly used to prevent new RNA synthesis and to evaluate the half-life and stability of mRNAs. We therefore tested its potential effect on our cells. Similar to temozolomide treatment, iGSCs were highly resistant to actinomycin D treatment, with significantly higher survival rates (data not shown). Salinomycin has been reported to selectively target CSCs 25. We therefore used salinomycin to compare the sensitivity of iGSCs and parental GBM cells, as an indirect measure of stem cell-ness. We treated both iGSCs and parental GBM cells with increasing doses of salinomycin for 2 days. As shown in Figure7B, iGSCs were highly significantly sensitive to salinomycin compared with GBM cells (*P* < 0.01).

## Discussion

Understanding the origin and maintenance of GBM CSCs is a major step forward in the development of novel and effective therapeutic strategies to prolong patient survival. In this study, we found that upon dedifferentiation with certain factors, patient-derived GBM cell lines acquire stem cell-like features (iGSCs), exhibiting self-renewal and multilineage differentiation. Compared with parental cells, they exhibited a more resistant phenotype to temozolomide, a first-line chemotherapeutic agent for GBM treatment, while becoming highly sensitive to salinomycin.

Acquisition of the stem cell state is linked to inactivation of RTKs and their major downstream pathways. Although EGFR and its downstream Akt and MAPK pathways have been linked to the pathogenesis of GBM 23,26,27, in our study these pathways and many other kinases were suppressed. It may be possible that suppression of EGFR expression would reverse the GBM cells towards the stem cell state since a recent study showed that EGFR inhibition induces a subgroup of cells that has high Oct4, Nanog and Klf4 expression levels 28. This subgroup had higher tumourigenic potential and resistance to therapy 28. iGSCs were primarily in a dormant state in accordance with significant EGFR, Akt and MAPK down-regulation. This may indicate that CSCs have certain activated internal factors and pathways driving their growth independently of external factors. This is supported by a previous study in which GBM stem cells were shown to proliferate independently of exogenous mitogens 2. It may be possible that EGFR and its downstream pathways are activated in differentiated tumour cells to promote their growth and survival, because CSC-related pathways are inactivated upon differentiation.

Oct4, Nanog and Sox2 have been linked to abnormal growth and oncogenic transformation 15,16, and emerging data suggest their role in GBM CSCs as well. Sox2 was shown to be activated in accordance with β-catenin in stem cell-like GBM cells, conferring resistance to radiation 13. In another study, Oct4 and Nanog were up-regulated in radioresistant GBM stem cells 28. It is yet unclear, how these factors promote tumourigenesis and resistance to treatment. Because they are vital for acquisition and maintenance of stem cell features, it is highly likely that these factors have effects on self-renewal and differentiation of CSCs. Recent studies indicated that they regulate CSCs through interaction with key pathways involved in neural development such as NOTCH, Wnt/β-catenin and SHH 13,29,30.

EphB4 is a member of the ephrin receptors, the largest subgroup of RTKs. Through cell–cell interaction, EphB4 receptor becomes activated by its ligand, ephrin B2, leading to regulation of vascular and neural development 31,32. On ELISA analysis, we identified hyperactivity of this receptor in iGSCs. Because EphB4 is involved in neural development, it may be possible that it also has an effect on the regulation of glioma CSCs. In fact, aberrant activity of the EphB4 receptor has recently been shown to stimulate tumour growth and vascularization in different kinds of tumours, including glioma 33–36. Furthermore, NOTCH activation was shown to regulate ephrin B2 expression and stimulate EphB4 receptor and ephrin B2 ligand interaction, leading to angiogenesis 37. Further studies would help elucidate the role of the EphB4 receptor in CSCs.

The key CSC pathways (NOTCH and Wnt/β-catenin) also exert regulatory functions on neural stem cells. Our results indicate that, as expected, both pathways became activated in iGSCs. The NOTCH signalling pathway is known to regulate neural stem cells by promoting self-renewal and inhibiting differentiation 38,39. Likewise, this pathway was shown to be active in glioma CSCs as well 12,40. NOTCH activation stimulates tumour growth, increases stem cell-like colonies and confers resistance to therapy 12,41. According to a recent study, NOTCH activation may be linked to CD133 positivity, as NOTCH inhibition resulted in loss of CD133^+^ colonies 40. Similarly, we also showed that in addition to NOTCH1 activation, transformed cells acquired a CD133^+^ state. CD133^+^ cells have been shown to exhibit stem cell properties with better colony formation and higher resistance to chemotherapy and radiotherapy 3,7,42. Because the functional significance of CD133 is still unclear, these features of CD133^+^ cells may be because of activated NOTCH1.

Aberrant β-catenin activity is a poor prognostic factor for GBM 43. Emerging data on Wnt/β-catenin signalling indicate that this pathway participates in the self-renewal and maintenance of GBM stem cells as well as radioresistance 13,44. We have shown that in iGSCs both β-catenin and Axin2 were activated. Although Axin2 is known as a canonical Wnt suppressor, an increasing number of studies have shown that it promotes oncogenic activity rather than functioning as a tumour suppressor 45,46. In addition, gene silencing of Axin2 results in diminished β-catenin activity and reversal of the epithelial-mesenchymal transition (EMT) process 46,47.

In conclusion, upon dedifferentiation of patient-derived GBM cells, transformed cells acquired stem cell features with activation of developmentally key pathways such as NOTCH1 and Wnt/β-catenin. Although the signalling pathway changes were not similar in iGSC1 and iGSC2, we did not see significant differences in iGSC stem cell-like behaviour or drug resistance. Because Oct4, Nanog and Sox2 were utilized for transfection of GBM cells, further elucidation of their role in the regulation of these pathways may help us understand CSC physiology and develop potential therapeutic interventions aimed at differentiating tumour cells to render them more sensitive to chemotherapy or other standard agents.

## Supporting Information

**Figure S1** Multilineage differentiation of iGSC1 detected with immunocytochemistry; Tuj-1 for ectoderm, GFAP for neuronal, GATA4 for endoderm and SMA for mesoderm.

**Figure S2** Neuroprogenitor cell (NPC) formation. Microscopic images of NPC1 and NPC2.

**Figure S3** Receptor tyrosine kinase (RTK) activity.

**Figure S4** Immunostaining for CD44 and ALDH1A1.
