# Supplementary figures and images for: Dedifferentiation of patient-derived glioblastoma multiforme cell lines results in a cancer stem cell-like state with mitogen-independent growth
Source: J Cell Mol Med. 2015 Mar 19;19(6):1262–72. doi: 10.1111/jcmm.12479 (PMC4459842; doi:10.1111/jcmm.12479)

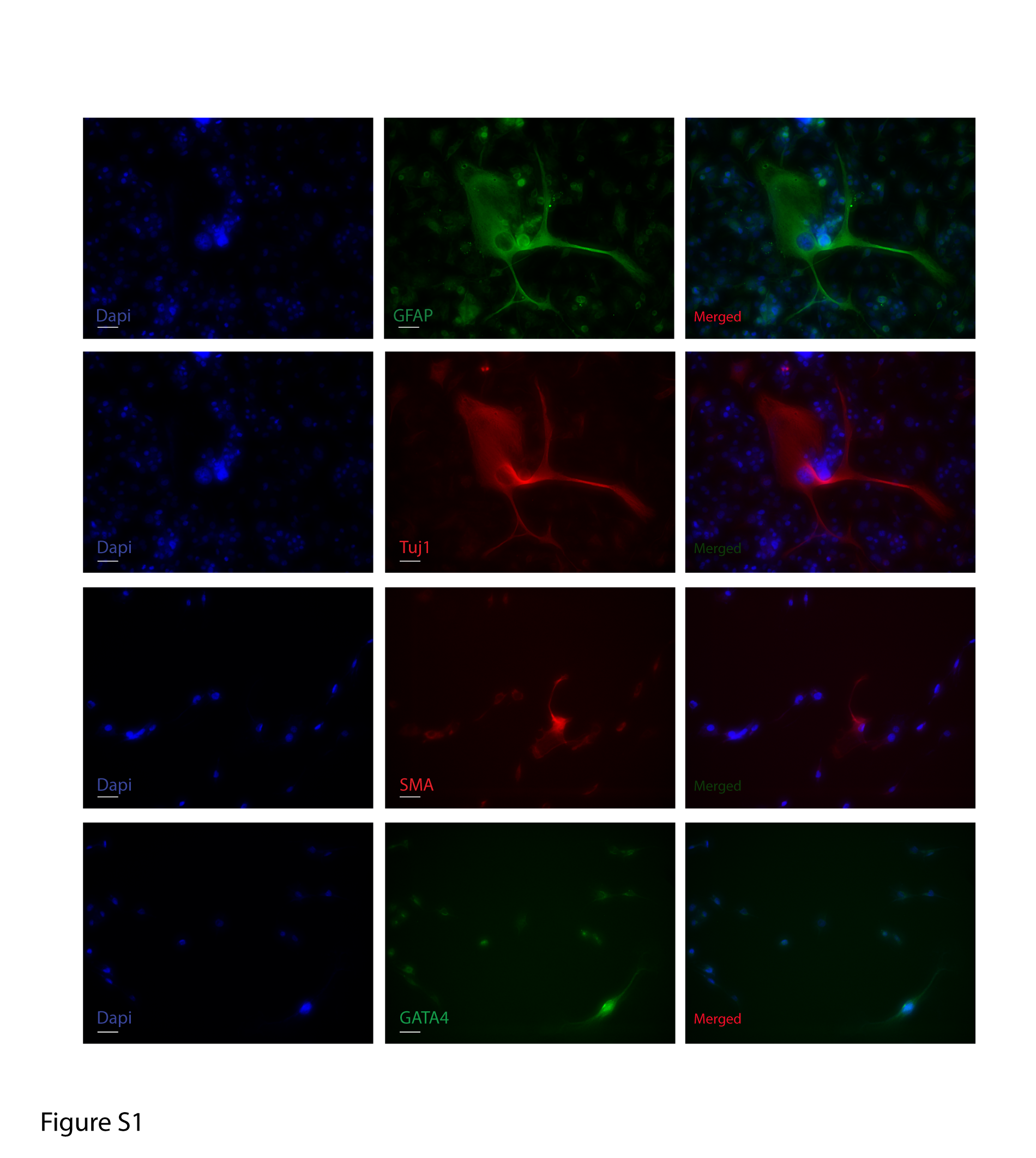

Supplement: Supplementary file 1 [file jcmm0019-1262-sd1.tif]

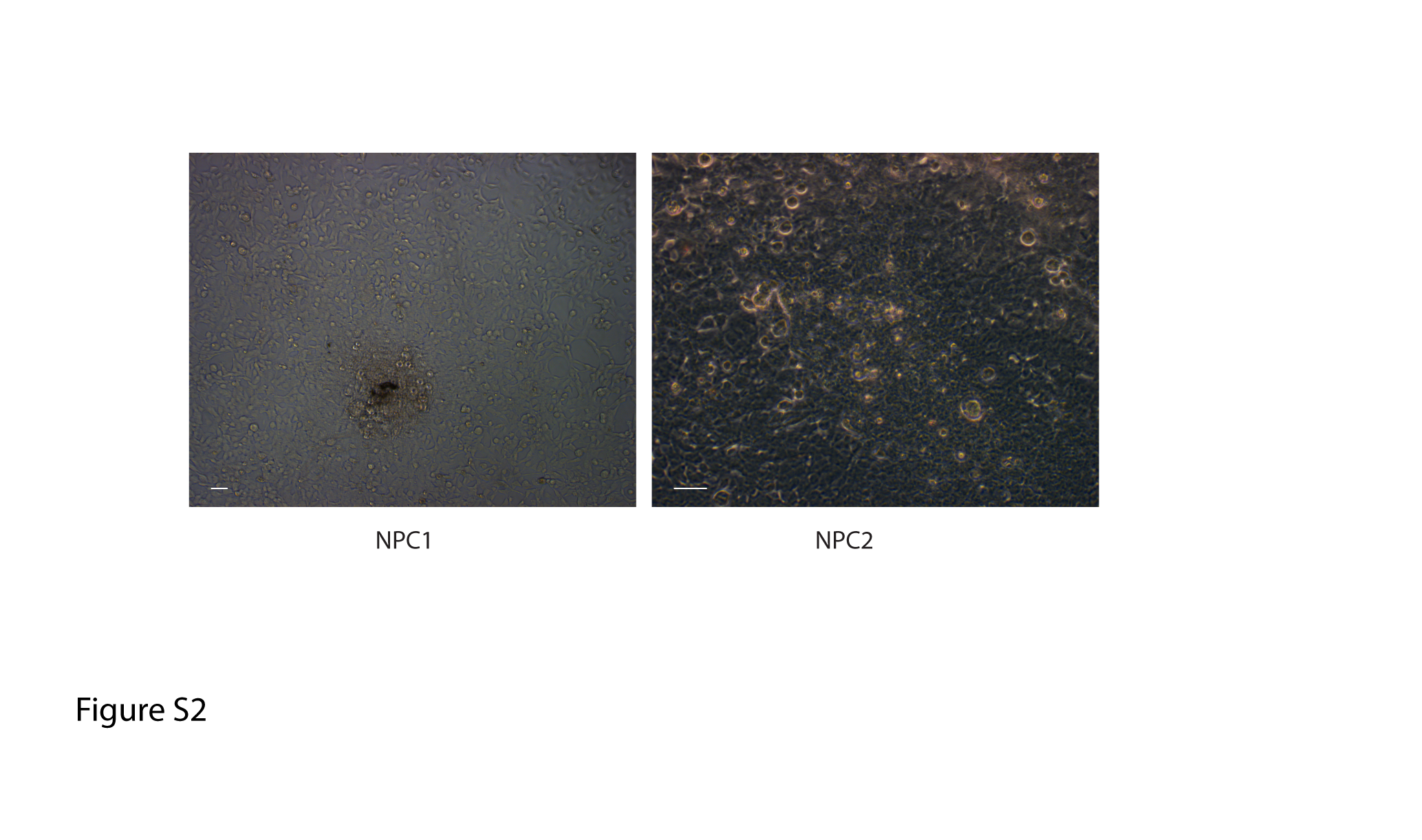

Supplement: Supplementary file 2 [file jcmm0019-1262-sd2.tif]

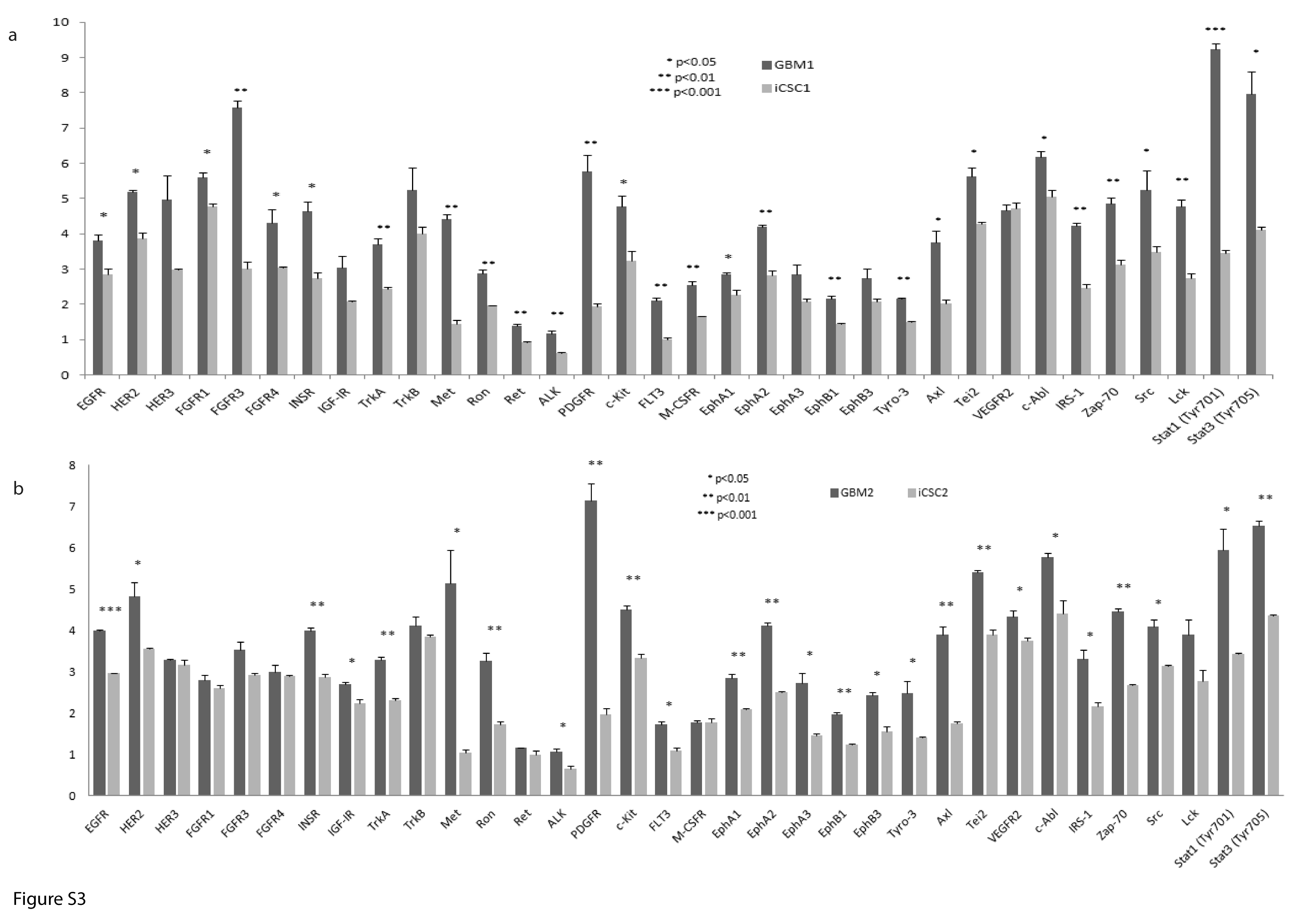

Supplement: Supplementary file 3 [file jcmm0019-1262-sd3.tif]

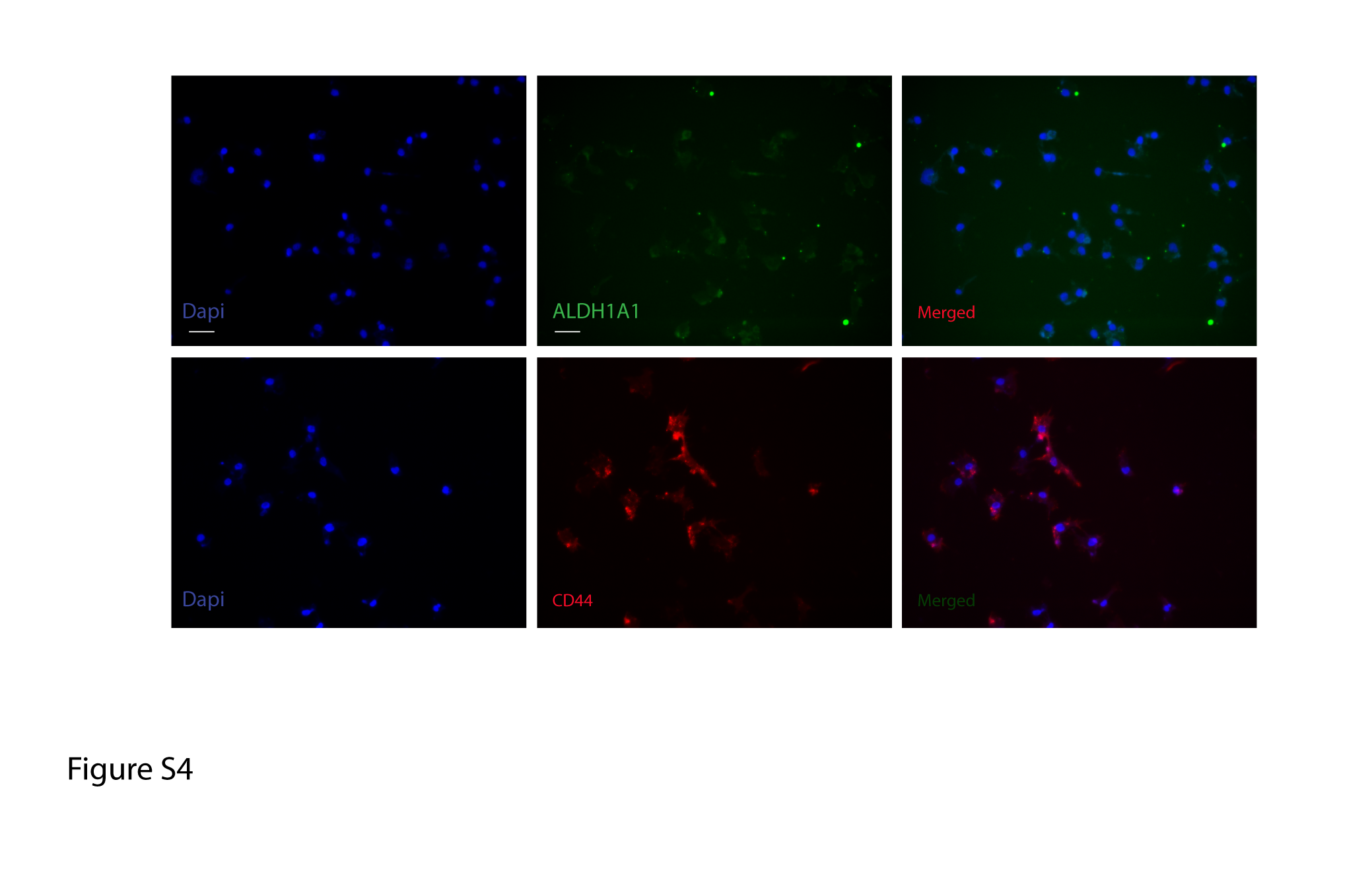

Supplement: Supplementary file 4 [file jcmm0019-1262-sd4.tif]
