# Supplementary material for: Dedifferentiation of patient-derived glioblastoma multiforme cell lines results in a cancer stem cell-like state with mitogen-independent growth
Source: J Cell Mol Med. 2015 Mar 19;19(6):1262–72. doi: 10.1111/jcmm.12479 (PMC4459842; doi:10.1111/jcmm.12479)
Supplement: Supplementary file 5 [file jcmm0019-1262-sd5.docx]

**Supplementary Figure Legends**

**Fig. S1** Multilineage differentiation of iGSC1 detected with immunocytochemistry; Tuj-1 for ectoderm, GFAP for neuronal, GATA4 for endoderm and SMA for mesoderm. Dapi was used for nuclear staining. Scale bar: 100 µm

**Fig. S2** Neuroprogenitor cell (NPC) formation. Microscopic images of NPC1 and NPC2. Scale bar: 100 µm

**Fig. S3** Receptor Tyrosine Kinase (RTK) activity. (A) and (B) ELISA-based phosphorylation status analysis of major RTK pathways revealed significant activity loss in iGSCs such as PDGFR, STAT1 and 3

**Fig. S4** Immunostaining for CD44 and ALDH1A1. iGSCs are positive for both CD44 and ALDH1A1. Dapi was used for nuclear staining. Scale bar: 100 µm
